# Mapping the perceived impacts of a social innovation program on women’s agency and life satisfaction

**DOI:** 10.3389/fsoc.2025.1527841

**Published:** 2025-02-14

**Authors:** Dina Tbaishat, Lina Qtaishat, Jannik Joseph Eggerman, Catherine Panter-Brick, Rana Dajani

**Affiliations:** ^1^Information Systems and Technology Management Department, Zayed University, Dubai, United Arab Emirates; ^2^Department of Impact Research, Taghyeer Organization, Amman, Jordan; ^3^Conflict, Resilience and Health Program, MacMillan Center for International Studies, Yale University, New Haven, CT, United States; ^4^Jackson School of Global Affairs, Yale University, New Haven, CT, United States; ^5^Department of Anthropology, Yale University, New Haven, CT, United States; ^6^Department of Biology and Biotechnology, The Hashemite University, Zarqa, Jordan

**Keywords:** agency, social innovation, life satisfaction, learning, reading, volunteering, system level simulation

## Abstract

**Introduction:**

Cross-cultural research measuring how women perceive their sense of agency and catalyze social innovation has been limited. We conducted a mixed-methods study to learn about women’s agency and life satisfaction, while evaluating the perceived benefits of a social innovation program (*We Love Reading*), in the UAE which, in 2022, launched a nationwide reading promotion strategy.

**Methods and results:**

We implemented the Sense of Agency (SoA) scale and Cantril Ladder of Life Scale with a sample of 78 female Emirati students, then conducted two Fuzzy Cognitive Mapping (FCM) sessions with 13 respondents. The FCM sessions helped to develop local definitions of agency and life satisfaction, map causal relationships, and run scenarios to identify program benefits. This highlighted 6 core dimensions of personal and relational agency—ability, control, strength, authority, freedom of action, and responsibility. *We Love Reading* boosted several aspects of agency and life satisfaction.

**Discussion:**

Our findings suggest that *We Love Reading* can help change mindsets and meet a fundamental policy goal related to reading habits and knowledge empowerment in the Arab World. They show the need for mapping causal reasoning in systematic ways, taking into account different dimensions of agency in environments where social innovation can flourish.

## 1 Introduction

Research measuring how women perceive their sense of agency and catalyze social innovation has been limited. Scholars have noted a lack of empirical work investigating how agency manifests itself in social innovations, defined as “new forms of cooperation that lead to new ideas on a local or regional level,” aimed at “addressing social challenges, satisfying human needs, empowering people, and changing social relations” (Wirth et al., 2023, pp. 3–4; Moulaert et al., 2005). Reviewing the proliferation of terms in fields of social entrepreneurship, social enterprise, and social innovation, Phills et al. (2008, p. 43) argued that the latter is the best construct for understanding social change: social innovation “blossoms where sectors converge,” embracing the exchange of ideas, shifts in roles and relationships, and the integration of public, private and nonprofit sectors, and creating social value to benefit society as a whole (Phills et al., 2008, p. 43). Agency is fundamental to social innovation, because it is creates new resources and capacities for transformational change. However, the scope of cross-cultural research on women’s perceived agency in social innovation is limited, especially in the Middle East region, despite notable interest in how women articulate a sense of agency, tackle social challenges, and positively impact society.

A good first step is to better understand, from the perspectives of women themselves, what a sense of agency and life satisfaction means to them, and how their agency, as power to originate actions, is causally related to social interventions. In this study, we measured women’s agency, noting the challenge of understanding this concept in the social sciences (O'Hara and Clement, 2018). We then mapped how women depicted local constructs, viewed systems of knowledge, and identified the benefits of a specific intervention.

We focused attention on a program (*We Love Reading*) striving for social innovation, one that empowers women to become leaders in their communities through promoting learning and reading across generations. To-date, this program has been implemented in 65 countries, reaching a diverse range of communities in rural and urban settings. In 2023, *We Love Reading* was introduced to female university students in the United Arab Emirates (UAE), in line with a governmental initiative to cultivate nationwide reading habits in order to catalyze educational, economic, cultural, and social change in Emirati society. Our research team sought to evaluate the perceived impacts of *We Love Reading* participation in terms of Emirati women’s sense of agency and life satisfaction, after they had initiated new reading-aloud behaviors intended to foster community engagement. Efforts to understand how women perceive what impacts their core agency, how they judge the quality of life around them, and how they catalyze change in society are critical to scholarly research and program evaluation.

### 1.1 Reading as social innovation

The *We Love Reading* program constitutes training volunteers to hold read-aloud sessions to children in their neighborhoods, using books that are age-appropriate, attractive, content neutral, and in the native language of the child (Mahasneh et al., 2021). Volunteers who complete the program, called *We Love Reading* Ambassadors, are invited to join an online network to help track and support their reading activities. In addition to promoting the experience of reading, the program aims to empower *We Love Reading* Ambassadors to become leaders in their communities, raising awareness on issues ranging from promoting health to taking care of the environment. By creating positive social change, these volunteers are potential social innovators. The program has won multiple awards and, to-date, trained more than 8,000 people to become *We Love Reading* Ambassadors globally, reaching over half a million children through 160,250 reading sessions and the distribution of 270,900 books. The program’s core philosophy is focused on empowering people to be change-makers in their own communities and to catalyze cultural change through reading and skill development. The training sessions emphasize storytelling techniques, reading aloud practices, personal agency, and social responsibility. The trainees learnt about the importance of reading for fun, how to read aloud, how to foster the love of reading, and how to choose a book for their libraries. After training, the participants received a certificate, and proceeded with reading activities, on a volunteer basis, accommodating reading practices whenever best suits their own timeline. Month-by-month follow-ups, conducted online or through WhatsApp, help monitor how often women engage in read-aloud activities. Beyond storytelling skills and accreditations, *We Love Reading* aims to foster a change in mindset amongst those who volunteer to pursue the program, promoting ways to think “I can do this” with regard to making change in their communities as social innovators.

The United Arab Emirates (UAE) provides an interesting context for *We Love Reading* implementation, given key priorities in governmental agendas and the private sector to promote the habit of reading and to foster intellectual growth in UAE society. Nearly a decade ago, the government declared 2016 as the UAE Reading Year, launching the country-wide National Literacy Strategy in light of concerns that reading practices were very limited, for either learning or pleasure, even amongst educated sectors such as university students (Zoubeidi et al., 2018). In 2022, the UAE launched a National Reading Strategy, which established “binding frameworks for all government agencies in the educational, community, media, and cultural sectors to establish reading among all groups of society in different age groups. [This sought] to enshrine reading as an integral part of the country’s social fabric” (Emirates News Agency [Wakalat Anba'a al Emarat], 2022). One specific leadership initiative has been the launch of the “Reading Month,” every March, as an ongoing movement to cultivate the habit of reading as a fundamental part of society’s development. The initiative specifically encourages individuals to volunteer a few hours of their time to reading for the elderly, sick, children, and those who are unable to read for themselves. As reported at the launch event, the UAE Minister of Culture and Youth stated that “The Reading Month […] is closely linked to the efforts of the UAE to establish a knowledge-based economy for the country’s progress and the well-being of its citizens and prepare for the future” (Emirates News Agency [Wakalat Anba'a al Emarat], 2022). The initiative aims to establish reading as a habit among 50 percent of Emirati adults and 80 percent of school students by the year 2026. Similar efforts to encourage reading are found in many Arab countries, including the “We Love Reading Initiative, which was launched in Jordan and has spread to Egypt, Saudi Arabia, State of Palestine, Tunisia, Lebanon, Iraq, Qatar and United Arab Emirates” (Arab Reading Index-ARI, 2016, p. 7). These initiatives represent national efforts in multiple Middle Eastern countries to promote a reading culture in society and drive change aimed at cultivating intellectual growth in society.

### 1.2 A sense of agency and life satisfaction

The construct of agency has long been debated and theorized within the cognitive and social sciences (Bandura, 2001, 2006; Burkitt, 2016), but has received renewed attention given its importance for understanding how one might promote learning and social innovation across populations. As articulated by Bandura (2001, p. 2), “to be an agent is to intentionally make things happen by one’s actions.” Thus “the power to originate actions” is key to personal agency (Bandura, 2001, p. 6; Code, 2020, p. 2). Bandura argued that four core features were essential to social cognitive dimensions of human agency. These were: intentionality and forethought (essential to planning ahead of time, as one moves through the life course), self-reactiveness (i.e., awareness and self-evaluation in formulating goals), and self-reflectiveness (i.e., perceived self-efficacy in one’s capabilities to adapt, change, and have impact). Other social theorists have emphasized that human agency is a highly relational phenomenon, emerging through social interactions across time and space, rather than found within the personal capacity of autonomous, independently-minded individual (Burkitt, 2016).

In the Arab world, scholars have highlighted that agency is multi-dimensional and unfolds in context-specific ways—calling for deeper understanding of construct meanings and more rigorous data collection efforts (Qutteina et al., 2019, p. 33; Yount et al., 2016, p. 1173). For example, in qualitative work with young Qatari students (*n* = 24), Qutteina et al. conducted cognitive interviews to understand intrinsic and instrumental agency—defined as a belief in oneself and as the ability to make choices—and to capture women’s interpretations of gender norms, decision-making, and freedom of movement. For her part, Pham (2013) expressed skepticism regarding a theorization of agency that centers “on freedom as well as on conscious self, and world transformation” (p. 42). Drawing from Saba Mahmood’s stories from the women’s mosque movement in Cairo, Egypt, Pham (2013) argued that women’s agency does not always align with a “singe desire for autonomy,” but within a “politics of piety” manifested through fortitude and steadfastness (p. 37), as women seek to reconfigure their traditions in society. Published work in the Arab world has considered women’s agency in relation to negotiating gender norms, patriarchy, Islamic piety (Kandiyoti, 1988; Kandiyoti et al., 2019) and lived religiosity (Duderija et al., 2020), as well as agency in relation to political representation (Welborne, 2016), workforce participation (Ramadan, 2022), involvement in social movements for democratization, civil society, and citizenship (Moghadam, 2013), and empowerment after the Arab Spring (Shalaby and Moghadam, 2016). This body of work discussed the interactions between agency and social structures and women’s role in creating social change. It largely followed Kabeer’s (1999) conceptualization of women’s empowerment as encompassing three dimensions: resources, agency, and achievement (p. 3). Significantly, Kabeer (2024) defined agency as “the capacity for thoughts and actions embedded in social actors” (p. 16) and placed women’s agency at the heart of a story about “how social change happens” (p. 3) to transform a nation.

To-date, despite rapid changes in the fabric of society, there is little scholarly work on women’s agency in the UAE. However, there is interest in studying gender norms, women’s empowerment, and attitudes to entrepreneurship, especially among the young and educated Emiratis (Alibeli, 2015; Kargwell, 2012; Majumdar and Varadarajan, 2013; Naguib and Jamali, 2015; Allagui and Al-Najjar, 2018). For example, Alibeli (2015, pp. 109–110) emphasized that while Emiratis live in a culturally conservative society, the UAE government has adopted policies aimed at “empowering women” via legislation, free higher education, and employment opportunities. Women’s empowerment is one of the UAE’s nation-branding strategy, given a “modernizing agenda that has highly publicized women’s roles in society, as leaders, educators, entrepreneurs, businesswomen, or ministers” (Allagui and Al-Najjar, 2018, p. 68). For their part, Majumdar and Varadarajan (2013, p. 289) characterized the UAE as having “a generation of young, educated women” able to positively interact in the changing social structure of an “innovation-driven economy.” But for many scholars, it remains “an open question what types of actors perform what types of activities in what type of agency, and how agency evolves throughout the social innovation processes” engendering regional transformation (Wirth et al., 2023, p. 2).

A sense of agency (SoA), more specifically, refers to “the subjective perception of being an agent” (Hurault et al., 2020, p. 1) when initiating a specific action in the world. It is related to an awareness of one’s attitudes and mental state, as well as awareness of one’s feelings of responsibility (Bart et al., 2023, p. 1). In this study, we followed the work of scholars interested in core agency, namely, a general belief in one’s agency, as distinct to a belief about one’s perceived success in obtaining a specific outcome. Specifically, for survey implementation, we used a self-reported measure that targeted a “cross-situational experience of agency, as distinct from how it unfolds in a specific experimental task or for a specific experience of agency” (Tapal et al., 2017, p. 7). This measure – the Sense of Agency Scale (SoAS)—has been implemented in English, French, German, and Hebrew, and validated with population samples of young people, mainly well-educated, and primarily female. Tapal et al. (2017) developed the 13-item scale in English and Hebrew, for use among a body of students (*n* = 236) at the University of Haifa, aged 24.3 years (SD = 3.6) and 75.80% female. Hurault et al. (2020) translated and validated the SoAC for implementation among French-speaking respondents (*n* = 517), aged 23 years, mostly students and 83.17% female. Lastly, Bart et al. (2023) validated the scale in a sample of German-speakers (*n* = 516), aged 28.47 (SD 10.48) and 64.92% female, across several European countries. Scholars have thus implemented this survey instrument as a useful measure of core agency - a cross-situational (rather than situation-specific) belief about having agency – among student populations across different social contexts. Importantly, the SoAS in intended to measure one’s own agency beliefs, rather than culturally-transmitted constructs such as notions of free will, determinism, or self-efficacy.

Life satisfaction, in contrast to agency, is a relatively straightforward concept. In the social sciences, it has been measured by the Cantril Ladder of Life Scale (Cantril, 1965), representing a subjective judgment of one’s life at the present time. Respondents are asked to visualize their life in terms of a ladder with 10 rungs, ranging from the worst possible life (0) and the best possible life (10), to help assess the degree to which they view themselves as achieving their overall goals (Kahneman and Deaton, 2010). This measure of life satisfaction has generated datasets in Gallup World Poll surveys in more than 150 countries (Helliwell et al., 2023). In the UAE, specifically, Cantril has been used to assess life satisfaction in relation to: demographic predictors such as age and marital status, social background predictors such as education, employment, economic predictors such as annual household income, and psychosocial characteristics including community-based experiences and social support (Lambert et al., 2020). In Jordan, Cantril was chosen as an outcome measure, along with psychological empowerment, to evaluate associations with the size and composition of women’s social networks (Eggerman et al., 2023) and to test program impacts of *We Love Reading* (Panter-Brick et al., 2024a).

### 1.3 Research strategy

Our study goals were to understand how women conceptualized agency, life satisfaction, and the perceived benefits a social innovation program (*We Love Reading*) in the Emirati context. We conducted a mixed-methods study to learn about women’s agency and life satisfaction while evaluating the perceived impacts of the program. We followed an explanatory sequential design (Creswell, 2009, pp. 194–195): namely, we developed an Arabic-language survey to measure levels of core agency and life satisfaction, then engaged in a participatory form of data collection (Fuzzy Cognitive Mapping) to translate respondent-led knowledge into cognitive maps suited to model system change. In the following sections, we detail the methodology, present main results, discuss their relevance, and make recommendations.

## 2 Methods

The study received full ethical approval from the Research Ethics Committee at Zayed University (Ethics Application Number: ZU23_072_F). The study began with a survey to measure the sense of agency and life satisfaction among a sample of Emirati women. Target participants were female students at Zayed University, within the campuses of Dubai and Abu Dhabi. Recruitment was done through classroom visits, poster distribution on campus, online campus announcements, and LinkedIn advertisement. There were no financial incentives for survey participation. While the research team initiated a follow-up survey to test *We Love Reading* program impacts, the number of respondents was too small to enable analyses of pre/post intervention data. Instead, We Love Reading Ambassadors were invited to convene in small discussion groups to engage with a participatory mapping approach to interrogate construct meanings and program benefits. After mapping sessions, simple gifts such as books and cosmetics were offered as appreciation of the time respondents spent in group discussion. All participants gave written informed consent.

### 2.1 Survey

Survey data collection was conducted in December 2023. Two female research assistants from Zayed University were hired for the purpose of recruiting participants and data collection. Survey instruments were chosen by team members (DT, RD, LS) to measure women’s socio-demographic background, sense of agency, and life satisfaction. Psychometric scales were translated into Arabic, back-translated into English, and piloted to ensure face validity. The Arabic-language survey, administered in Qualtrics, consisted of self-reported responses and took about 15 min to complete. Table 1 details items measuring the Sense of Agency and life satisfaction, in both English and Arabic.

**Table 1:** Main survey instruments in English and Arabic: sense of agency and Cantril’s Ladder of Life Scale.

	Arabic: **مقياس الشعور بالملكية**
English: sense of agency^a^	
I am in full control of what I do I am just an instrument in the hands of somebody or something else My actions just happen without my intention I am the author of my actions The consequences of my actions feel like they do not logically follow my actions My movements are automatic—my body simply makes them The outcomes of my actions generally surprise me Things I do are subject only to my free will The decision whether and when to act is within my hands Nothing I do is actually voluntary While I am in action, I feel like I am a remote-controlled robot My behavior is planned by me from the very beginning to the very end I am completely responsible for everything that results from my actions	لدي تحكم كامل بما أفعله أنا مجرد أداة في يد شخص أو شيء آخر أفعالي تحدث بدون نيتي لذلك أنا صاحب أفعالي عواقب أفعالي تبدو وكأنها لا تتبع منطقياً لأفعالي ذاتها. حركاتي تلقائية – جسدي يقوم بفعلها ببساطة نتائج أفعالي تفاجئني بشكل عام. الأشياء التي أفعلها تخضع فقط لإرادتي الحرة القرار بأن أتصرف و متى أتصرف هو بين يدي لا شيء أفعله هو في الواقع اختياريّ (طوعيَ) و أنا أقوم بفعل الأشياء، أشعر بأنني رجل آلي يتم التحكم به عن بعد سلوكي هو مخطط له من قِبلي من البداية و حتى النهاية أنا مسؤول تماماً عن كل شيء ينتج عن أفعالي.
Cantril’s Ladder of Life Scale (life satisfaction)^b^	**مقياس الرضا عن الحياة**
Please imagine a ladder with steps numbered from zero at the bottom to ten at the top. Suppose we say that the top of the ladder represents the best possible life for you and the bottom of the ladder represents the worst possible life for you. If the top step is 10 and the bottom step is 0, on which step of the ladder do you feel you personally stand at the present time? **Choose from 0 to 10**	لطفاً، تخيل سلماً بدرجات مرقمة من صفر في الأسفل و حتى عشرة في الأعلى، افترض بأن أعلى السلم يمثل أفضل حياة محتملة لك و أن أسفل السلم يمثل أسوأ حياة محتملة لك. ؟إذا كانت أعلى درجة هي 10 و أسفل درجة هي0، على أي درجة من السلم تشعر بأنك تقف عليها حالياً **اختر من 0 إلى 10**

a English version from Hurault et al. (2020) and Tapal et al. (2017). Responses are elicited on a 7-point Likert scale 1–7 *(1 = Strongly disagree, 7 = Strongly agree). Items 1, 4, 9, 12 and 13 constitute the sub-scale on positive agency (SoPA). Items 2, 3, 5, 6, 7, 10, and 11 constitute the sub-scale on negative agency (SoNA).*

b After Cantril (1965).

The Sense of Agency (SoA) scale is a 13-point instrument, with responses evaluated on a 7-point Likert scale (1 = strongly disagree, 7 = strongly agree). Developed by Tapal et al. (2017), using explanatory and confirmatory factor analyses, the scale has a two-factorial structure, tapping into positive and negative factors. The two sub-scales measure having control (a sense of positive agency, SoPA) and feeling existentially helpless (a sense of negative agency, SoNA). Thus, SoPA represents the judgment of “feeling in control of one’s body, thought, and environment,” whilst SoNA represents the judgment of feeling “existentially helpless” (Tapal et al., 2017, p. 8). In the original Hebrew-language study, two sub-scales were weakly correlated, and showed good psychometric properties and two-month test–retest reliability (Tapal et al., 2017). In a subsequent German-language study (Bart et al., 2023), the sub-scales also showed good internal consistency. In our study, the Arabic-language scale also showed good internal consistency (Cronbach’s *α* = 0.79 for SoPA and α = 0.86 for SoNA).

The Cantril Ladder of Life Scale asks respondents to visualize their goals and achievements as spanning a ladder with 10 rungs. It features a 1-item response ranging from 0 (worst possible life) to 10 (best possible life). It has been found a useful indicator of life satisfaction in Middle Eastern countries such as the UAE (Lambert et al., 2020) and a valuable outcome variable for program evaluation in Jordan (Panter-Brick et al., 2024a).

Following the survey, a quarter of the cohort (*n* = 24) volunteered to receive the *We Love Reading* training. Training sessions were online, in the form of recorded videos on a dedicated portal, and offered over a period of 2 months (January–February 2024). Such online training has been developed in Arabic and English languages, since 2018, for training volunteers globally. While a follow-up survey was initiated, the numbers of respondents were too small to statistically compare women who did vs. did not engage in *We Love Reading* as originally planned.

### 2.2 Fuzzy cognitive mapping

Fuzzy Cognitive Mapping (FCM) is a methodology used to generate a ‘mental landscape’ representing stakeholder knowledge—a ‘cognitive map’ which respondents generate to visualize a network of variables and assess the strength of causal relationships. This method is helpful to elucidate complex constructs, to make inferences from respondent-led data, and to learn from different stakeholder groups regarding what may drive system change (Barbrook-Johnson and Penn, 2022; Panter-Brick et al., 2024b). It is a participative method of data collection that lends itself to a thematic analysis of belief systems and can be translated into quantitative models of system change (Gray et al., 2012). When modeling scenarios of change, the methodology rests on ‘fuzzy’ quantitative measures, rather than exact measurement of the relative weight of connections in maps drawn by respondents. The visual representation of relative impacts, across variables of interest, is nonetheless valuable. Thus, in FCM sessions, respondents identify variables, draw connections, and specify relative weights to causal relationships, ranging from −1 to +1 in value. A modeling tool, called Mental Modeler, is available online and free of charge, to generate the map representing stakeholder knowledge and to compute the strength of impact linking variables within that knowledge systems. Table 2 details the steps taken to facilitate FCM discussion, visual representation, and analysis.

**Table 2:** Fuzzy cognitive mapping: qualitative, visual, and quantitative steps in the methodology.

Map generation		
Step 1	Preparation	Participants are invited to sit in a semi-circle. The facilitator explains the methodological approach, demonstrating how maps will be visualized, in real time, using the online Mental Modeler platform. Research questions are specified (e.g., what does a sense of agency and life satisfaction mean for you, and what are the causal factors of influence?).
Step 2	Identifying system variables	Discussion begins with participants identifying key variables (e.g., dimensions of agency and life satisfaction) and factors of influence (e.g., *We Love Reading* program). The facilitator encourages participants to present different perspectives and balance viewpoints. The discussion is audio recorded to aid subsequent analysis.
Step 3	Identifying connections	Participants are invited to build a mental landscape, iteratively, with the map visible to all (e.g., projected on a wall). They connect the different variables with causal links (unidirectional arrows) and identify whether these connections are positive or negative (enhancing or diminishing other variables). They also identify sets of variables that conceptually go together (e.g., agency) with different colors on the map.
Step 4	Assigning weights	Participants assign relative weights to links drawn on the map, using values ranging from −1 (very negative) to +1 (very positive) to quantify the strength of influence. Each incoming and outgoing arrow will be assigned an in-degree or out-degree value. The facilitator ensures the map reflect a collective viewpoint, encouraging participants to assign weights as fuzzy, rather than exact, measures of relative importance. The final map, generated by the software and agreed upon by all participants, is saved digitally as a Mental Modeler file.
Map analysis		
Step 1	Thematic analysis	The verbatim text is transcribed for thematic analysis, conducted by the research team to identify key concepts, patterns, insights, and context. The research team identifies key examples from lived experience and ways of reasoning.
Step 2	Visual graph analysis	Visual analysis aims to identify the main variables and key relationships in the system map. Graph theory indices, generated by Mental Modeler, calculate the sum of the weights assigned to incoming and outgoing links connecting the variables. Variables with high degree centrality (with highest total sum of weights) indicate that they are significant to the participants and play key role in the system. Driver variables (with highest out-degree values for outgoing arrows) are most consequential for system change. The maps can be visually represented using the Gephi software, to better visualize the centrality of key variables and the most consequential drivers.
Step 3	Scenarios	The research team runs what-if scenarios, maximizing or minimizing the relative strength of specific factors of interest, to quantitatively model system change. For example, the weights assigned to a specific variable (*We Love Reading*) are activated to their maximum value (+1), to predict impacts on key outcomes (e.g., agency). The scenarios are saved digitally in Mental Modeler, and can also be represented in Canva.

We implemented Fuzzy Cognitive Mapping with *We Love Reading* respondents (*n* = 13) in April–May 2024. Using WhatsApp messaging for easier communication, it became clear that respondents were best recruited to two sessions, one in the Dubai campus, the other in the Abu Dhabi campus, to accommodate student time and workloads. Study goals were re-iterated at the start of each mapping session (by DT and RD, both university professors from the Arab region). The sessions were led by LQ, an Arab researcher and project manager experienced in facilitating FCM methodology using the online tool known as Mental Modeler. They began with the questions: “what does life satisfaction mean to you? what does a sense of agency mean for you?” Respondents were asked what factors influenced these concepts, and as the discussion unfolded, to visually map variables and inter-connections for all to see. Each session lasted 3 h and was audio-recorded (in Arabic).

### 2.3 Analyses

Survey data were elf-reported. We excluded respondents who gave incomplete answers on 3+ items of Likert-scale outcome variables. We examined all variables for data distribution, after item-by-item imputation for 1–2 missing items of Likert-scale data (7.58% of cases). To evaluate the strength of expected associations, we ran pairwise correlations for SoPA, SoNA, and Cantril scores. To characterize the sample, we used binary variables for single/married status, university-level education, low/high household income, and previous/no volunteering community experience.

The FCM analysis proceeded in three steps (Table 2): thematic analysis of participant narratives, visual examination of maps, and quantitative simulation of system change. In step one, we used inductive thematic analysis (Braun and Clarke, 2006) to understand how respondents discussed and theorized a sense of agency and life satisfaction. Two team members (LQ, RD) reviewed the Arabic-language recordings of focus group sessions, then grouped together the types and meanings of different constructs, selecting examples of verbatim discussion to serve as specific illustrations. All team members convened using videoconferencing to clarify the relevance of constructs and their connections. This helped to tabulate specific instances of how women discussed agency and life satisfaction. In step two, we examined the maps, identifying the unidirectional arrows that represented causal influence and noting their relative (positive or negative) strengths of influence. Here we used Gephi, another online tool, to visually represent maps and scenarios according to their relative importance. In step three, we used the Mental Modeler software to create scenarios, analyzing how the system responded to maximizing or minimizing the relative strength of specific factors of interest.

## 3 Results

### 3.1 Survey

Seventy-eight students, all students at Zayed University, completed the survey. As shown in Table 2, they averaged 22.65 (SD 4.27) years in age; 88.46% were single. Half the sample (47.44%) had completed a higher-level degree, such as a Bachelor’s or Master’s degree. Just over half (58.97%) came from households with a monthly income over 20,000 AED (equivalent to ~USD 5,500); for comparison, the average monthly income of an Emirati family is 72,241 AED (Dubai Statistics Center, 2016), equivalent to ~USD 19,700.

As a group, Emirati students scored higher on the positive than the negative sense of agency subscale, averaging 5.25 (SD 1.05) points for SoPA and 3.09 (SD 1.30) points for SoNA, respectively. The correlation between the two subscales was not significant (r = −0.12). For Cantril, Emirati students average a middle-of-the-ladder score for life satisfaction (average 6.53, SD 2.64). As expected, the correlations between Cantril scores and the Sense of Agency sub-scales were significant (Cantril and SoPA: r = 0.27, *p* = 0.03; Cantril and SoNA: −0.35, *p* = 0.003).

### 3.2 Thematic analysis

Women were asked to express what agency (الملكية) and life satisfaction (الرضا عن الحياة) meant to them. As shown in Table 3, respondents identified several, interrelated meanings of agency, thematically grouped into six dimensions: *ability, control, strength, authority, freedom,* and *responsibility.* Fundamentally, a sense of agency meant the perceived *ability* to make changes, make decisions, and express thoughts: in the words of respondents, agency was “the ability to change our lives for the better” and “be able to take the decision to develop ourselves,” and it meant that “you can express your thoughts.” Agency also rested on a sense of *control*—being in control of every aspect of life, including behaviors, decisions, choices, and relationships. On a psychological level, women also expressed a sense of agency as the *strength* to have “determination and persistence” to get through difficult life circumstances, as well as self-confidence (“the trust that you can do it”), and a sense of self-reliance and independence (being “reliant on themselves […] to have full agency over the things around them”). Importantly, women clarified that a sense of agency is not limited to the individual self. They saw agency as a form of *authority* and power over others: for example, mothers had “agency arising from authority” over their children, teachers had authority over their students, and governors had power over their citizens, an authority based on maturity, experience, or privilege. In relational spaces, to have agency rested on *freedom*, the “freedom to do anything” and “to have the full right to refuse or agree with no [fear of] consequences.” Importantly, according to respondents, a sense of agency was coupled with a sense of *responsibility*: it is “to be accountable for one’s actions,” “being responsible for taking my decisions, either positive or negative, and bear the consequences.”

**Table 3:** Characteristics of survey respondents.

Age (mean, SD)	22.65 (4.27)
Marital status, Single (n, %)	69 (88.46%)
Education level (n with BA/Masters, %)	37 (47.44%)
Household income (>20,000 AED/month)	46 (58.97%)
Sense of agency (SoA)	
SoPA, mean (SD)	5.25 (1.05)
SoNA, mean (SD)	3.09 (1.30)
Life Satisfaction (Cantril)	6.53 (2.45)

13 women did not report their monthly income (AED, Emirati Dirhams).

SoPA, Sense of Positive Agency; SoNA, Sense of Negative Agency.

Women also identified six types of agency: over oneself, over thoughts, over decisions and actions, over health, over one’s fate or future, and over relationships. They described agency over oneself as “being able to take the decision to develop oneself” and “not to be influenced by others,” or not “having to follow the opinion of the majority instead of their own.” Women discussed their agency over thoughts in terms of the ability to “express” and “produce a better quality of thoughts.” Agency over decisions and actions was associated with “autonomy,” “being aware and able to control,” and responsibility. Agency over health concerned both physical and mental well-being, such as the choice “to follow a diet” or the “ability to solve one’s mental issues” and seeking help if needed. Agency over the future included personal goals, career aspirations, education, and choices. Finally, agency over relationships was about “the way [women] impact other people,” but also “having stronger relationships with [people and] engage with them.” Specifically, women stated that “people who have agency over their relationships usually put boundaries;” they have “the freedom to cut and stop” the negative and “toxic” relationships, as well as the choice to “strengthen [positive relationships] and intensify communication.”

Turning to life satisfaction (Table 4), respondents distinguished 8 facets of this construct. To be satisfied with one’s life entailed a state of *acceptance* and *contentment.* Acceptance meant that a person would “not to compare oneself with others,” but accept her circumstances, problems, decisions, mistakes, and fate. Contentment meant that a person did not have everything that mattered but could be “satisfied with what’s available.” This state of mind was “different than settling” for one’s stage in life; on the contrary, it meant “accepting and actually enjoying what you have without wanting more.” Life satisfaction meant that women could be better prepared for adapting and coping with life, “instead of giving up” on life. It rested upon gratitude, a keen awareness of “the things happening in life,” and a “*reconciliation*” with the challenges of life. Importantly, women saw life satisfaction as *peace of mind*: “not thinking about tomorrow, about the future, and not to think about the things I did before.” It embodied a belief in destiny or fate, “believing that everything that happened is the best thing that could happen to you,” even illness or missed opportunity. In their discussion, women also differentiated a sense of satisfaction with oneself, with one’s mental health status, and with what Allah had provided. Specifically, they highlighted the need “to love oneself, to accept oneself, and not to be influenced by others.” They recognized that “when mental health is good, everything around will fall into place,” meaning that people were able to “take the right decisions…[and] not neglect themselves.” In terms of what had been provided by Allah, women referred to *Arzaq*, a term that was encompassing of all of Allah’s bounty: “it is not only money, relationships are *Arzaq*, good feelings are *Arzaq*… good people are *Arzaq,* as well as the career.” *Arzaq* embraced being married, having children, gaining knowledge through education, being in good health, and having good relationships with friends and family.

**Table 4:** The meanings of agency, as discussed by Emirati women in fuzzy cognitive mapping sessions.

**Ability (القدرة)**	**Authority (السلطة)**
*Ability:* “That we have the ability to change our lives for the better.” *Making decisions:* “To be able to take the decision to develop ourselves.” *Expression of thoughts:* “Agency means that you can express your thoughts.”	“As a mother I control my children’s directions because I have the authority and they are not mature by experience… it’s agency arising from authority.” *Authority as a form of power:* “Power is also having authority.” “Power is the power to change.”
**Control (السيطرة والتحكم)**	**Freedom (الحرية)**
*Control:* “To control everything in my life.” “I have an agency over my behaviors, my directions, over my decisions or choices.”	*Freedom:* “That you are not forced on anything… you have the freedom to do anything.” “To have the full right to refuse or agree with a certain thing with no consequences.” “No constraints… nothing external is impacting the decisions you are taking.”
**Strength (القوة)**	**Responsibility (الشعور بالمسؤولية)**
“Is to have determination and persistence.” *Strength as a form of power:* “Power is strength or perseverance… being able to get through things and being resilient.” *Strength as self-confidence:* “The trust that you can do it.” *Self-reliance & Independence:* “Self-reliance is independence” … “When someone is reliant on themselves, it means that they have full agency over the things around them.”	“To be accountable for one’s actions.” “Being responsible for taking my decisions, either positive or negative and bear the consequences.”

In brief, women discussed agency and life satisfaction as multi-faceted constructs. They articulated the personal dimensions of agency: one’s perceived ability to change life, one’s perceived control over behaviors and decisions, and one’s perceived strength as the power to persevere. Importantly, they also articulated the relational dimensions of agency as lived through social interactions—perceived authority or power to change other people, perceived freedom to refuse or agree with no consequences, and responsibility for taking actions and bear consequences. They interpreted life satisfaction in terms of one’s personal journey—cultivating feelings of acceptance, contentment, and peace of mind—as well as one’s spiritual journey, marked by faith in destiny, adapting positively to life situations, and practicing awareness, gratitude, and reconciliation. In the next section, we turn to examine what factors causally influenced women’s sense of agency and life satisfaction.

### 3.3 Visual analysis

**Figure 1:**
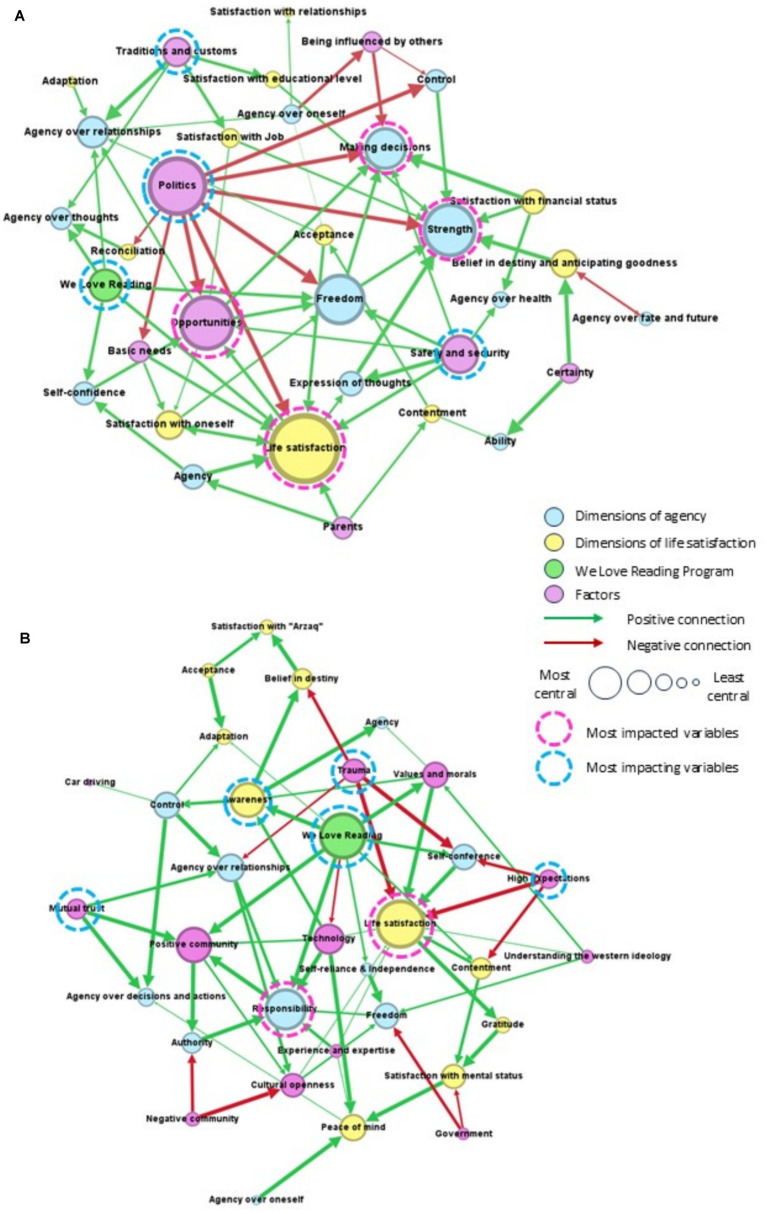
Maps of agency and life satisfaction drawn by women during fuzzy cognitive mapping sessions, on the **(A)** Dubai and **(B)** Abu Dhabi student campus. The main constructs identified in group discussion are shown by circles, the sizes of which represent centrality. The directional arrows (positive and negative) represent causal influences. The most impacting variables are consequential drivers of system change (**A**—Politics, Safety and security, Traditions and customs, and *We Love Reading*; **B**—Awareness, High expectations, Mutual trust, Trauma, and *We Love Reading*).

The ‘mental maps’ for agency and life satisfaction, constructed by women on the Dubai and Abu Dhabi campus, are shown in Figures 1A,B. The main constructs identified in group discussion are shown by circles, the size of which reflects the centrality of each variable, namely, its relative importance in the system (as defined in Table 2). The most impacting variables, considered to be consequential drivers of change, are denoted by a blue circle. The most impacted variables, considered to be receptors of change, are denoted by a pink circle. The directional arrows (positive and negative) represent causal influences, and the thickness of links indicates the relative strength of causal relationships. Our analysis focused attention on the constructs respondents deemed important (the larger circles) and the consequential drivers of systems change (impacting variables, or blue circles). We noted that in both maps, women saw the concept of life satisfaction as one relatively large, central variable. By contrast, they represented the concept of agency as multiple variables (e.g., freedom, making decisions, responsibility, and strength) in the system. In terms of causal influences, women were quick to discuss political and social factors, not just individual-level experiences. Figure 1A showed four consequential drivers (politics, safety and security, traditions and customs, and *We Love Reading*), while Figure 1B showed five (awareness, high expectations, mutual trust, trauma, and *We Love Reading*).

Politics was the most consequential driver of change in Figure 1A: it was the larger impacting variable (blue circle), with as many as eight outgoing, red arrows (negative connections). It had direct, negative impacts on life satisfaction, opportunities (“to create something new”), and four dimensions of agency (control, freedom, making decisions, and strength). Referring to current events, women remarked: “*Humanity fails over and over again to be human, and it all goes back to politics.*” Women discussed global politics, governments, freedom, as well as safety and security as causal influences.

The other three driver variables exerted positive influences, as shown by green arrows. For one, “safety and security” impacted life satisfaction, opportunities, as well as four dimensions of agency (agency over health, expression of thoughts, freedom, and making decisions). Also positive was the influence of Emirati “traditions and customs,” seen by respondents to “*support education and jobs*” [for women], as well as agency over thoughts and over relationships, through supporting traditional family gatherings. Women saw that society would have a positive influence when it “supports and lift up people” and “encourages cultural openness,” but could exert a negative influence when it “limits freedom, sense of responsibility and autonomy,” when it “weakens authority,” and when it “fears cultural openness.” Women discussed with pride their Emirati traditions and customs, explaining that “it supports their education, jobs… [and] empowers women.” In their view, Emirati traditions promoted “openness to other countries… coexistence and tolerance.” The third impacting variable, in this map, was the *We Love Reading* program, with five (green) arrows directed to enhance life satisfaction, agency over thoughts, agency over relationships, freedom, and self-confidence.

*We Love Reading* was the most central driver variable in Figure 1B: it made positive impacts on system change, with seven direct causal links (green arrows) drawn to heighten awareness, contentment, responsibility, self-confidence, a positive community, values and morals. Other positive drivers were awareness and mutual trust (as denoted by outgoing green arrows), while negative influences were attributed to high expectations and trauma (as denoted by outgoing red arrows). Thus women reflected that higher levels of awareness (of “the things happening in life”) strengthened a belief in destiny, their adaptation to life circumstances, and their sense of agency and control. They saw that mutual trust among society members contributed to a more positive community and enhanced one’s agency over relationships, decisions, and actions. By contrast, high expectations, without achieving expected results, negatively impacted levels of contentment. Traumas, which included the loss of a loved one, domestic violence, bullying, accidents, and the wider political climate, would “*cause trust issues*,” decreased agency over relationships, and negatively affect a belief in destiny and anticipation of goodness in life.

Importantly, in both maps, *We Love Reading* was seen as a positive driver of system change. Specifically, engagement in *We Love Reading* boosted personal and relational agency, as well as life satisfaction. On a personal level, it enhanced self-confidence, self-reliance, and independence, through which women “*were able to leave an impact*” on the younger generation. Women believed the program promoted their agency over thoughts and ideas, asserting that “*reading allows people to think from another perspective*.” In terms of enriching social relationships, women gave the following example: “*people might not know how to communicate with others and might have social anxiety, but through reading with [close] family members, then the bigger family and the community, they break the barrier*.” Breaking the barrier referred to making friends, to networking, to relationships between people. Importantly, women saw that *We Love Reading* positively impacted their sense of freedom, because “*it gives the space to explore more, and to be free with imagination and the type of stuff one wants to do*.” However, it also placed a higher responsibility on them; for example, they needed to “*look at the content of the book before [the child] reads it… there are books that do not positively impact the child’s imagination; the child imagines each word they receive. It is a responsibility to read the book before a child does*.” Notably, engagement with *We Love Reading* increased their life satisfaction and contentment levels because of the “*positive impact made in society*.” It also enhanced their awareness and adaptation to circumstances, by “*filling the age gap and learning what children love*.” Women saw that, through reading aloud to children, they could reinforce positive cultural values and contribute positively to the community.

### 3.4 Scenarios

**Figure 2:**
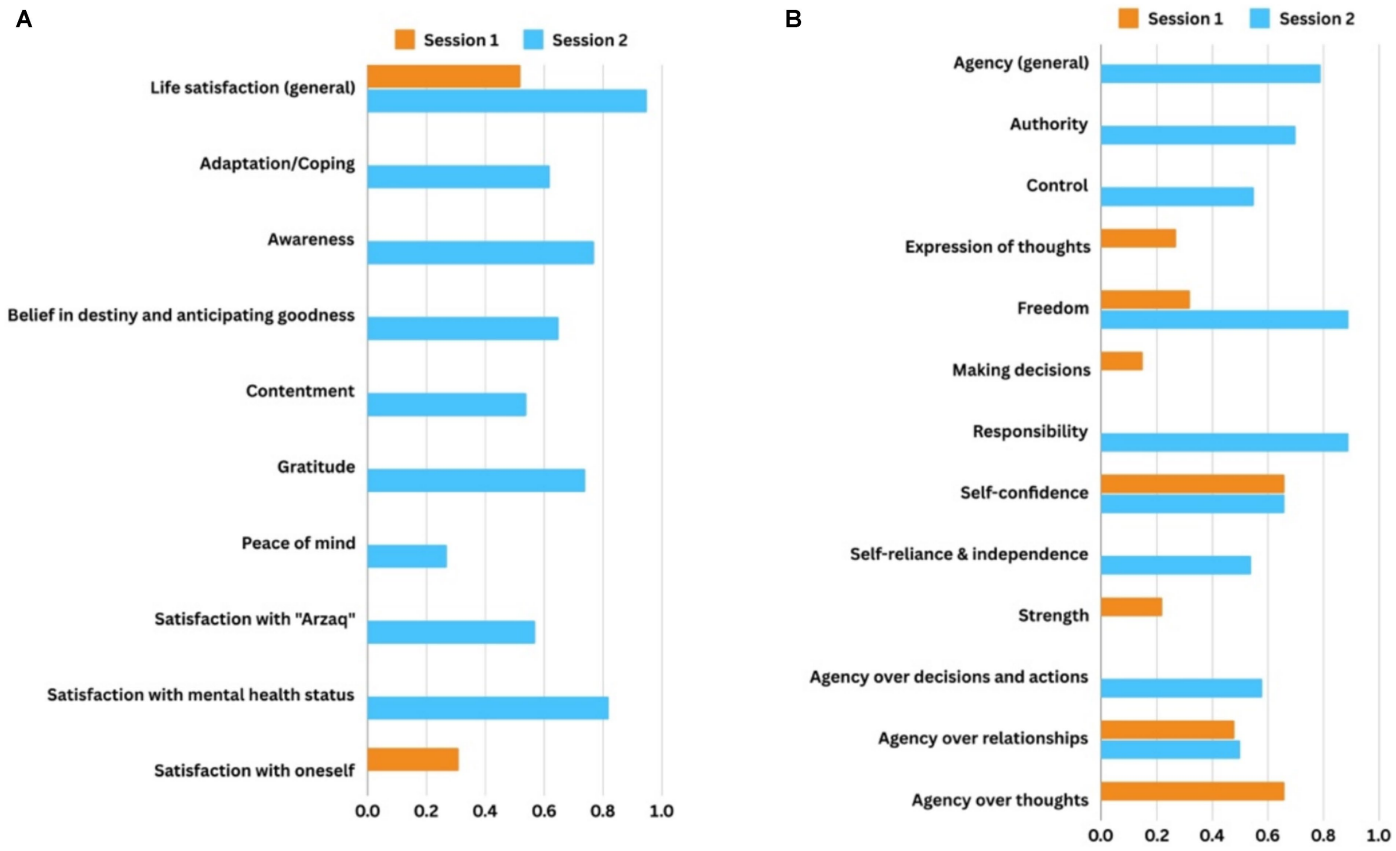
Scenarios modeling the impacts of *We Love Reading* on women’s **(A)** agency, and **(B)** life satisfaction. The driver variable (the *We Love Reading* program) was given a maximum value (+1) for the weight of causal connections in scenario simulation.

Our final step was to run scenarios to model system change. We ran “what-if” scenarios to explore how engagement with *We Love Reading* affected the outcome variables that women had mapped in the system. Using the Mental Modeler program, we specifically activated the *We Love Reading* variable: namely, we maximized (to a maximum value of +1) the relative weight assigned to *We Love Reading* within the system. Figure 2 shows two scenarios modeling the impacts on dimensions of (a) agency and (b) life satisfaction. Based on the causal connections mapped by respondents, the scenarios predict the systems impacts of *We Love Reading*.

In Figure 2, scenario A, we see a cascade of effects on dimensions of agency. In both session 1 and session 2, women identified that *We Love Reading* engagement would increase agency over relationships, perceived freedom, and self-confidence (the variables with dual horizontal bars). This means that *We Love Reading* increased agentic notions of freedom (“to have the full right to refuse or agree … with no consequences”) and self-confidence (“the trust that you can do it”), such that women could be better equipped for negotiating relationships, taking action, and having an impact on society. In session 1, engagement in *We Love Reading* also promoted agency over thoughts, self-expression, and inner strength, especially because reading allowed people to think from new perspectives. In session 2, *We Love Reading* promoted agency (as a general concept), and agentic dimensions of authority, control, responsibility, self-reliance and independence.

In Figure 2, scenario B, we see that *We Love Reading* engagement would increase levels of life satisfaction in general (the variable with dual horizontal bars). Women also discussed how *We Love Reading* would increase satisfaction with oneself (session 1) and other facets of life satisfaction (adaptation, awareness, belief in destiny, contentment, gratitude, peace of mind, satisfaction with oneself, with one’s mental health status, and with *Arzaq* provided by Allah, session 2). These scenarios make explicit several pathways or mechanisms by which the program influence specific dimensions of the constructs under consideration.

## 4 Discussion

Our study aimed to learn about women’s agency and life satisfaction in the Middle East, in region where, as social actors, their actions can help knowledge transformation and processes of social innovation take hold. In the Middle East, specifically, concepts of agency and empowerment, as well the mechanisms of change, are contested or poorly defined (Qutteina et al., 2019; Panter-Brick et al., 2024a). We evaluated the perceived impacts of *We Love Reading*, a social innovation program that trains women to hold read aloud sessions to children in their neighborhoods, as well as empower them to promote social change through a culture of learning and volunteer service. We did so with a lens to understand the potential for promoting a culture of reading and social innovation in the UAE, a country transitioning from post-oil dependence to a “knowledge economy” (Lambert et al., 2020; Emirates News Agency [Wakalat Anba'a al Emarat], 2022). We structure this discussion in terms of two questions: first, what did we learn about perceived *We Love Reading* impacts and about women’s agency in the Emirati context, and second, what are the merits of survey methods and visual, scenario-building approaches to knowledge representation?

### 4.1 Program benefits and women’s agency

In this study, what did we learn about perceived *We Love Reading* impacts and, more specifically, about the construct of agency? Through participatory discussion and visual mapping, women identified that *We Love Reading* increased agentic notions of freedom and self-confidence, as well as agentic negotiation of social relationships. They specified that engagement with *We Love Reading* would boost agency (as a general concept), authority over others, control over life, responsibility, self-reliance, agency over thoughts, and agency over decisions and actions. Engagement with *We Love Reading* also increased life satisfaction, because of the positive impact women could make in society. Such beneficial changes over multiple dimensions of agency and life satisfaction were evidenced in Figure 2, in which the driver variable (*We Love Reading*) was given its maximum value (+1) in scenario simulation.

It is instructive to discuss these results in light of findings from a *We Love Reading* program evaluation conducted with Syrian refugee women in Jordan (Panter-Brick et al., 2024a). That study used a randomized controlled trial to test program impacts, as well as cognitive mapping methodology to visualize causal connections to life satisfaction, empowerment, and agency. Notably, *We Love Reading* boosted “cultural empowerment,” (Panter-Brick et al., 2024a, Figure 3) creating opportunities for learning and social interactions outside the home, thus enabling women to “have a role in society.” It also boosted agency (as a general concept), agency over self, agency over time, and agency over people (Panter-Brick et al., 2024a, Figure 4). Syrian women spoke of empowerment as “ability” and “freedom to be act [and] be able to take her own decisions,” as well as “acquiring a right” (Panter-Brick et al., 2024a, Table 4). Living in low-income households, within a culturally conservative society where women’s behaviors were socially regulated and under the scrutiny of kin, Syrian refugees articulated empowerment as acquiring “the full right to act,” negotiating family support to counteract “negative traditions and customs” that confined women to the home. They spoke of cultural empowerment as opportunities “to learn, to study, to educate oneself, … to become productive in the community.” They differentiated between “agency over self” (being able “to control my opinions, my feelings”), “agency over people” (“how I impact people”), and “agency over life paths” (Panter-Brick et al., 2024a, p. 9). A subsequent study, engaging both Syrian and Jordanian women from resource-poor families, concluded that women “framed their access to decision-making power in ways that were highly relational, going beyond notions of individual-level agency, power, or control” (Panter-Brick et al., 2024b, p. 11).

Our study engaged a wealthy sample of young Emirati women, who commanded many material, economic, and educational resources in society. They held positive views of their life situation, averaging a Cantril score of 6.53 (SD 2.45) in the 10-step ladder of life satisfaction. Patterns in the global data (Gallup, 2009) suggest that high scores (>7) characterize respondents with who are thriving, rather than struggling (scores 5–7) or suffering (scores 0–4). Yet they articulated similar understandings of agency and life satisfaction as low-income, Syrian refugees whose Cantril score averaged 5.30 (SD 2.32) (Panter-Brick et al., 2024a). In both Jordan and the UAE, Arab women described life satisfaction as acceptance, adaptation, contentment, peace of mind, referring to a state of tranquility and ease. Both refer to lived religiosity, invoking their faith in destiny, their trust and gratitude in Allah, to tackle challenging life circumstances. When seeking to learning and working opportunities, both refer to strength, self-confidence, and “the full right” in decision-making, defining freedom as “the full right to refuse or agree with a certain thing with no consequences.”

**Figure 3:**
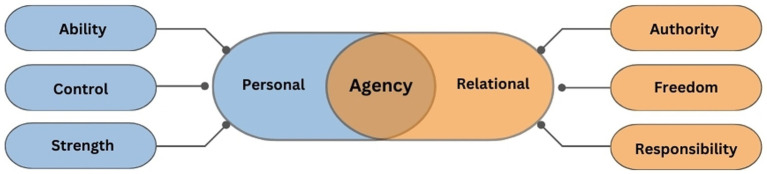
Core dimensions of women’s personal and relational agency. This visualization is based on the meanings of agency identified through participatory cognitive mapping methodology. For example, Agency was defined as the “ability to change our lives to the better” and “control over my behaviors, my directions, over my decisions or choices.” Agency as strength was defined as “to have determination and persistence,” encompassing the power of perseverance, self-confidence and self-reliance. Agency as authority was described as the power to change people’s life direction. Agency as freedom rested on having “to have the full right to refuse or agree with a certain thing with no consequences.” Agency as responsibility meant being “accountable for one’s actions.” We need to take into account different dimensions of agency when considering how and why social innovations can flourish.

We know from previous literature that because women’s agency is “exercised in different ways and in a number of different domains,” measuring multiple dimensions of agency is key to robust cross-cultural research (Hanmer and Klugman, 2016, p. 242). In this study, Emirati women described personal and relational agency as interrelated and nested within six core dimensions (thematically shown in Figure 3). These encompassed three dimensions of personal agency—*perceived ability, perceived control, and inner strength*. By ability, women meant being able to “change our lives for the better,” “take the decision to develop ourselves,” and “express your thoughts” (Table 3). By control, respondents meant having agency over their behaviors, decisions, and choices. By strength, women articulated having a form of “power” expressed through determination, persistence, perseverance, self-confidence, self-reliance and independence while navigating one’s life. In addition, women discuss three meanings of agency pertaining to their social interactions—*authority over authors, freedom of action, and responsibility* for decisions and actions. Authority was manifested in the “power to change” other people and their life direction. For example, in the Arab world, when women assume culturally-prescribed roles as mothers and home-makers, women’s authority is exercised through shaping the learning and reading opportunities of children. Women discussed agency as freedom in terms of having free will and independence from others, separating themselves from dense family and social ties that would influence employment, marriage, networking, and social standing. With both authority and freedom, came heightened responsibility, namely accountability in actions and social interactions.

These dimensions of agency echo those found in other Arab, Middle Eastern contexts. Thus Qutteina et al. (2019) highlighted, for Qatari students, perceptions of a woman’s intrinsic and instrumental agency as follows: “a belief in herself and awareness of her rights (power within)” as well as “her ability to affect her life by having power to make choices and to control acquired resources” (p. 34), especially power of decision-making and freedom of movement. For Egyptian women involved in the Islamic mosque movement, the Islamic concept of *sabr* (patience, fortitude, and steadfastness) was key to negotiating relationships and expressing collective agency within the constraints of divine fate (Mahmood, 2011; Pham, 2013). In this study, young Emirati students largely centered their discussions on notions of personal agency, yet also included agentic notions of having power over other people (authority), not being bound by other people (freedom), and developing accountability (responsibility). These are different ways to exercise agency, influencing how women may engage with addressing social challenges and social innovation.

### 4.2 Methodological approaches to knowledge representation

Scholars have noted that power as agency “is one of the most contested and ambiguous concepts in the social sciences” and that designing a tool to measure empowerment or agency across cultural contexts “presents daunting challenges” (O'Hara and Clement, 2018, pp. 111, 112). In the Middle East, psychometric research on women’s empowerment and agency has been limited (Yount et al., 2016), despite a call for deeper understanding of local knowledge pertaining to these constructs. By contrast, population surveys have reported that the Ladder of Life (Cantril) is a well-understood instrument, and culturally-relevant in the Arab world (Panter-Brick et al., 2024a, p. 10).

In this study, we implemented the Sense of Agency scale (Table 1), a 13-item measure designed to measure core agency (a general belief in one’s agency, irrespective of perceived success in a specific situation). While the Sense of Agency scale has been validated in other student populations, we did not have a sufficiently large sample to conduct factor analyses for psychometric validation. We see that SoA items tap constructs of perceived control (items 1 and 9), intentionality and forethought (items 2–7, 11–12), free will (items 8 and 10), and responsibility (item 13). With respect to a sense of control, free will, and responsibility, these items resonate with the core dimensions of agency identified by Emirati women (Figure 3). For example, SoA item 1 asks respondents to respond to the statement “I am in full control of what I do.” Emirati women declared that agency meant “to control everything in my life” and to have “agency over my behaviors, my directions, over my decisions or choices.” However, the SoA items omit the dimensions of agency as inner strength (the “power of perseverance”) and authority (the “power over people”).

Other scholars have chosen to measure agency as the ability to initiate and direct actions towards a specified goal. A good example is the Agency for Learning (AFL) scale, developed and validated with student populations in a Canadian university (Code, 2020). This work largely focused on individual agency, extending social cognitive theory “from the perspective of the learner in the context of learning” (Code, 2020, p. 3), a type of agency rooted in intention motivation, self-efficacy and self-regulation. In her work, Code tested a composite of existing instruments, including intentionality and forethought (28 items), self-regulation (11 items), and self-reflectiveness (11 items). In factor analysis, the AFL scale (42 items) measured agentic functioning along six dimensions of intentionality (goal planfulness and decision confidence), forethought (intrinsic and extrinsic motivation), self-regulation, and self-reflectiveness/self-efficacy. The scale was meant “to measure agentic capabilities as mediating factors between personal, environmental, and behavioral processes” (p. 3), to provide a more unified approach to the study of learning processes in individual and social settings.

While psychometric surveys are useful to predict how social actors will behave across space and time, they provide just one methodological approach to understanding the links between agency, for example, and social innovation. The fuzzy cognitive mapping methodology utilized in this study is well-suited to understand complex constructs and system change (Gray et al., 2012; Panter-Brick et al., 2024b). This approach is participatory: discussants apply their local knowledge to generate visual representations of variables of interest and evaluate their interconnections. The method helped us pinpoint how Emirati women went beyond the dimensions of agency theorized by social cognitive scientists, and provided original insights into local knowledge, causal reasoning, and potential scenarios of change.

What this fuzzy cognitive mapping methodology reveals, thematically, is that women attached importance to inner strength (‘power within’) and authority (‘power over others’), in addition to notions of perceived ability and control. It revealed, visually, a ‘mental map’ of causal connections and predicted the systemic impacts of a discrete intervention such as *We Love Reading*. Evaluating the perceived benefits of a reading aloud program, through the eyes of women who volunteer to read aloud in their community, is important for testing fundamental assumptions about local stakeholder knowledge and the transformative power of social innovation.

### 4.3 Study strengths and limitations

This study has two main strengths, as well as two main limitations. First, we developed an Arabic language translation of the Sense of Agency, a scale known to be psychometrically valid in student populations in several other countries (Tapal et al., 2017; Hurault et al., 2020; Bart et al., 2023). However, we did not have enough survey participants to conduct an exploratory factor analyses to confirm the two-factor structure of this measure, and we were not able to conduct pre-post analyses to quantitatively analyze *We Love Reading* impacts. Despite targeted recruitment efforts, including in-class recruitment, a relatively small number of Emirati women were willing to engage in survey measures.

Second, the study took a participatory, visual and semi-quantitative approach to understand perceived agency, life satisfaction, and program benefits. Respondents in fuzzy cognitive mapping sessions were enthusiastic about this way of understanding their life experiences, articulating many dimensions of local constructs under discussion, and clearly visualizing how an intervention could impact them. Due to the constraints of time and distance, only two mapping sessions were convened, which means that insights are limited to those of a small group of respondents. The method has some limitations: the maps do not describe all factors of influence; bias can arise, if some respondents are more vocal than others in group discussion, although this can be mitigated by a skilled facilitator; and hypothetical scenarios provide limited insights to potential systems change. Nonetheless, this methodology is useful to describe local knowledge systems: Sarmiento et al. (2020, p. 12) noted that fuzzy cognitive maps offer “soft models” of the way people reason and depict knowledge structures, while scenarios offer “a transparent and systematic way” to organize viewpoints across language, education, or culture, thus ensuring that “Western epistemological frameworks need not go unchallenged in intercultural settings.”

### 4.4 Recommendations

This brings us to make two recommendations. The first speaks to policymakers implementing new initiatives to bridge gaps of knowledge in the Arab World, where young people, including university students, have very limited reading habits (Zoubeidi et al., 2018), and where governmental and civil actors push to revive Arabic language, intellectual growth, and cultural heritage. As highlighted in a report produced by the Mohammed Bin Rashid Al Maktoum Knowledge Foundation (MBRF) and the United Nations Programme Development Nations (UNDP), reading is “undoubtedly one of the gateways to knowledge and development,” as well as “an essential means for empowering people and communities” (Arab Reading Index-ARI, 2016, p. 32). Thus the UAE has sought to articulate a National Reading Strategy that binds government agencies to establish reading as a behavioral and cultural habit in society (Emirates News Agency [Wakalat Anba'a al Emarat], 2022). A governmental strategy will often promote reading through formal education systems: for instance, the UAE recently won a UNESCO literacy prize for an e-learning platform providing Arabic-language education in schools worldwide (UNESCO News, 2022). However, it is also necessary to promote reading as a mindful activity, through volunteered practices embedded in family systems and local communities. Our first recommendation is thus to embed programs such as *We Love Reading* into family life, community initiatives, cultural habits, and educational systems. In Jordan, for example, *We Love Reading* has been successfully adopted by the Ministry of Culture through the launch of a national read-aloud campaign in 2022. The idea is that *We Love Reading* promotes a culture of reading, fosters early literacy, promotes lifelong learning, and involves family members in reading to children. It teaches the love of reading for pleasure, an important change in mindset (Mahasneh et al., 2021). Importantly, if Arabic language books are selected for reading aloud, it can promote Arabic language, a fundamental goal of the UAE National Reading Strategy.

The second recommendation speaks to scholars and practitioners interested in linking agency with social innovation. Our findings suggest that we need to take into account different dimensions of women’s personal and relational agency, when considering how and why social innovation can flourish. It would be fruitful for research to carefully map causal reasoning with diverse groups of stakeholders to better understand, in transparent and systematic ways, how diverse social actors see agency, social innovation, and system change. Importantly, social innovation entails “dissolving boundaries and brokering a dialogue between public, private, and nonprofit sectors” (Phills et al., 2008, p. 36). We found the Fuzzy Cognitive Mapping methodology helpful in making room for qualitative analyses of local constructs and visual analyses of stakeholder knowledge, as well as quantitative modeling of potential system change. This approach allows one to interrogate agency and social innovation in a range of contexts, and offer clarity on best practices, project design, and added value of program implementation (see Table 5).

**Table 5:** The meanings of life satisfaction, as discussed by Emirati women in fuzzy cognitive mapping sessions.

**Acceptance (التقبل)**	**Contentment (القناعة)**
“To accept what I have and not to compare myself with others.” “To accept circumstances.” “To accept the problem that I have.” “To accept decisions.” “To accept Allah’s destiny and fate.”	“I do not have everything… but I am satisfied and content with the things I have … it is not that I do not want to be better, but at this stage I am satisfied with what’s available.” “It is different than settling… it is more of accepting and actually enjoying what you have without wanting more, and think what you have is enough.”
**Adaptation/Coping (التكيف والتأقلم)**	**Gratitude (الامتنان)**
“Instead of giving up, I accept and adapt with the current situation until I get used to it.” “Even if my life is not the way I want it to be, I accept it and cope with it, to turn it in a positive way.”	“To be grateful for the experiences.” “Gratitude is being thankful.”
**Awareness (الوعي)**	**Peace of mind (راحة البال)**
“To be aware and understand the things happening around me and the things happening in life… and to know how to behave”	“To wake up in the morning not thinking about many things, not to think about tomorrow, about the future, and not to think about the things I did before…to be relaxed.”
**Belief in destiny and anticipating goodness (الخيرة والإيمان بالقضاء والقدر)**	**Reconciliation (التصالح)**
“Having faith in destiny and fate and believing that everything that happened is the best thing that could happen to you.”	“To reconciliate with life as we were friends… to accept it… and at the same time, I am looking forward for developing it.”

## 5 Conclusion

In this study, we argue that it is important to deepen cross-cultural knowledge on how women exercise agency to address social challenges, meet human needs, and contribute to social innovation. We conducted our research with female Emirati students in order to better understand the perceived impacts of an innovative read-aloud program on agency and life satisfaction. This is of particular importance given the context of the UAE which launched a national reading promotion strategy in 2022. We developed an Arabic-language translation of the Sense of Agency scale, and through fuzzy cognitive mapping methods, clarified how respondents understand and articulate local knowledge on constructs such as agency. Research on these topics has been limited, particularly in the Middle East. Women identified that the *We Love Reading* program boosted their agency across multiple dimensions of personal and relational agency. In this context, the six dimensions of agency were: ability, control, strength, authority, freedom, and responsibility. The fuzzy cognitive mapping approach demonstrated in this study offer a grounded, visual approach to localize knowledge—showing how women perceive agency and understand systems change. Their perspectives offer a deeper understanding on the potential of promoting a culture of learning and social innovation.

## Data Availability

The raw data supporting the conclusions of this article will be made available by the authors, without undue reservation.
